# A Retrospective Comparative Study of Sodium Fluoride Na^18^F-PET/CT and ^68^Ga-PSMA-11 PET/CT in the Bone Metastases of Prostate Cancer Using a Volumetric 3-D Radiomic Analysis

**DOI:** 10.3390/life12121977

**Published:** 2022-11-25

**Authors:** Kalevi Kairemo, Aki Kangasmäki, Srinivasan Cheenu Kappadath, Timo Joensuu, Homer A. Macapinlac

**Affiliations:** 1 Department of Theragnostics, Docrates Cancer Center, 00180 Helsinki, Finland; 2 Department of Nuclear Medicine, MD Anderson Cancer Center, Houston, TX 77030, USA; 3 Department of Medical Physics, Docrates Cancer Center, 00180 Helsinki, Finland; 4 Department of Imaging Physics, MD Anderson Cancer Center, Houston, TX 77030, USA; 5 Department of Medical Oncology and Radiotherapy, Docrates Cancer Center, 00180 Helsinki, Finland

**Keywords:** PSMA, prostate specific membrane antigen, sodium fluoride, positron emission tomography, prostate cancer, bone metastases, radiomics

## Abstract

Bone is the most common metastatic site in prostate cancer (PCa). ^68^Ga-PSMA-11 (or gozetotide) and sodium fluoride-18 (Na^18^F) are rather new radiopharmaceuticals for assessing PCa-associated bone metastases. Gozetotide uptake reflects cell membrane enzyme activity and the sodium fluoride uptake measures bone mineralization in advanced PCa. Here, we aim to characterize this difference and possibly provide a new method for patient selection in targeted therapies. **Methods:** The study consisted of 14 patients with advanced PCa (M group > 5 lesions), who had had routine PET/CT both with PSMA and NaF over consecutive days, and 12 PCa patients with no skeletal metastases (N). The bone regions in CT were used to coregister the two PET/CT scans. The whole skeleton volume(s) of interest (VOIs) were defined using the CT component of PET (HU > 150); similarly, the sclerotic/dense bone was defined as HU > 600. Additional VOIs were defined for PET, with pathological threshold values for PSMA (SUV > 3.0) and NaF (SUV > 10). Besides the pathological bone volumes measured with each technique (CT, NaF, and PSMA-PET) and their contemporaneous combinations, overlapping VOIs with the CT-based skeletal and sclerotic volumes were also recorded. Additionally, thresholds of 4.0, 6.0, and 10.0 were tested for SUV_PSMA_. **Results:** In group M, the skeletal VOI volumes were 8.77 ± 1.80 L, and the sclerotic bone volumes were 1.32 ± 0.50 L; in contrast, in group N, they were 8.73 ± 1.43 L (skeletal) and 1.23 ± 0.28 L (sclerosis). The total enzyme activity for PSMA was 2.21 ± 5.15 in the M group and 0.078 ± 0.053 in the N group (*p* < 0.0002). The total bone demineralization activity for NaF varied from 4.31 ± 6.17 in the M group and 0.24 ± 0.56 in group N (*p* < 0.0002). The pathological PSMA volume represented 0.44–132% of the sclerotic bone volume in group M and 0.55–2.3% in group N. The pathological NaF volume in those patients with multiple metastases represented 0.27–68% of the sclerotic bone volume, and in the control group, only 0.00–6.5% of the sclerotic bone volume (*p* < 0.0003). **Conclusions:** These results confirm our earlier findings that CT alone does not suit the evaluation of the extent of active skeletal metastases in PCa. PSMA and NaF images give complementary information about the extent of the active skeletal disease, which has a clinical impact and may change its management. The PSMA and NaF absolute volumes could be used for planning targeted therapies. A cut-off value 3.0 for SUV_PSMA_ given here is the best correlation in the presentation of active metastatic skeletal disease.

## 1. Introduction

Bone is the most common metastatic site in prostate cancer (PCa); about 90% of patients with metastatic-castration-resistant prostate cancer (mCRPC) have radiological evidence of bone metastases [1]. Men who develop metastatic disease are commonly treated with androgen deprivation therapy (ADT), which frequently leads to tumor regression [2,3]. The responses are typically transient, resulting possibly in tumor regrowth as castration-resistant disease [4]. The median overall survival of mCRPC is approximately two years [5]. Deaths from prostate cancer usually arise due to complications from skeletal metastases bone disease, unlike in many other cancers.

Prostate-specific membrane antigen (PSMA), which has two enzymatic functions (either folate hydrolase I or glutamate carboxypeptidase II, depending on their location in the human body), is a type II transmembrane protein. PSMA is highly expressed in prostate epithelial cells and strongly upregulated in prostate cancer. The PSMA expression levels are directly proportional to androgen independence, metastases, and the aggressivity of prostate cancer [6]. Thus, at present, PSMA is a promising molecular target for the diagnosis and therapy of metastatic prostate cancer [6]. There are multiple PET-tracers, which target PSMA [7]. A network meta-analysis can confirm the superiority [8] of the three most commonly used PSMA-radiotracers (^68^Ga-PSMA-11, ^18^F-PSMA-1007, and ^18^F-DCFPyl), particularly when compared to previous-generation choline-based tracers [8,9]. Prostate targeting PET tracers have prognostic significance, which have been recently reviewed systematically [10].

Sodium fluoride-18 (NaF) is a radiotracer that has been known for a long time. The Society for Nuclear Medicine and Molecular Imaging (SNMMI) has published guidelines for ^18^F -NaF PET/CT in the US [11], and there are national guidelines in Europe, e.g., in Denmark, ^18^F-NaF-PET is included as an alternative for screening skeletal metastases in prostate cancer [12]. Retrospective and prospective studies have demonstrated that the whole-body skeletal tumor burden on baseline ^18^F-Fluoride PET/CT is a powerful imaging biomarker for evaluating the prognosis of prostate cancer [13,14].

The uptake of ^18^F-NaF is reflected by bone remodeling and growth. Positive ^18^F-NaF PET/CT scans are associated with a high risk of skeletal metastases [12]. When circulating prostate cancer cells are transformed into bone marrow and deposited in the cortical bone, skeletal metastases are formed. Recently, an 8.3-year median survival has even been achieved with active experimental multimodal treatment approaches in advanced prostate cancer with bone metastases [15]. On the other hand, it is known (from a retrospective worldwide multicenter study) that the presence of skeletal metastases is a poor prognosticator of effective targeted prostate cancer treatments [16]. The aim of this study was to analyze the role of contemporaneous ^68^Ga-PSMA-11-PET, Na^18^F-PET, and CT imaging in advanced metastatic skeletal disease. We have presented the principle of this method and the initial results at the SNMMI Congress [17]; this is the full report of the retrospective analysis.

## 2. Materials and Methods

### 2.1. Patients

In this retrospective analysis, we included all those PCa patients who had both [^18^F]-NaF-PET and [^68^Ga]-PSMA-11-PET as a part of clinical staging on consecutive days as a part of their diagnostic program. Prostate cancers were diagnosed between 1996 and 2016 (Table 1). The patient recruitment was performed in the same manner as in [18].

#### 2.1.1. Group M

At Docrates Cancer Center in Helsinki, 14 new consecutive patients were analyzed between November 2015 and January 2017 without any further selection. Their ages varied from 54 to 79 years (66.2 ± 8.9 years), their Gleason scores (GS) from 5 to 9, and their initial PSA values from 6.7 to 968 µg/L; a total of nine patients had T3–T4 disease, four had T2 disease, and one had T1c disease (Table 1). All patients had skeletal metastases, and three of them had visceral metastases. The radiological TNM staging was performed using [^18^F]-NaF-PET (NaF-PET) and [^68^Ga]-PSMA-11-PET (PSMA-PET) in all patients; most (12 out of 14) of the patients also had pelvic MRI and whole-body diagnostic CT. These patients had more than five identifiable bone metastases from the PET studies (in order to confirm the skeletal metastatic disease); the criteria were similar to those published in [18]. Seven men had previous surgery (6 RP+1 TURP), 10 had been treated with radiotherapy, all 14 with androgen deprivation therapy (ADT), 12 patients with chemotherapy, three had received Sm-153-EDTMP therapy, and six Ra-223; six additionally received denosumab (Table 1) (Arabic numbers).

#### 2.1.2. Group N

At total of 12 control patients with prostate cancer had no skeletal metastases. They were the last 12 men studied during the same period as the M group patients without any further selection. Their characteristics are shown in Table 1 (Roman numbers). Their radiological TNM staging or restaging was performed using [^18^F]-NaF-PET and [^68^Ga] Ga-PSMA-11-PET on consecutive days. The men’s ages varied from 50 to 83 years (68.5 ± 10.1 years); GS from 6 to 9; initial PSA values from 0.3 to 36 µg/L. Seven of these patients had T3–T4 disease, one had T2 disease, and three had T1 disease (Table 1).

### 2.2. Imaging PET/CT Protocol

The patients were imaged for gozetotide [^68^Ga]-PSMA-11-PET/CT using Biograph TruePoint PET-CT (Siemens, Erlangen, Germany). Patients were injected with [^68^Ga]-PSMA-11; average activity was 350 MBq (range 210–400 MBq). Imaging was performed in two phases: early pelvic body imaging at an average time of 11 ± 4 min and late, whole-body imaging at an average time of 60 ± 5 min.

The whole body was imaged from the calvarium to the mid-thighs, as described in [18]. Then, (Na^18^F)-PET/CT imaging was performed at 60 min (range 58–76 min) as a whole-body imaging (from the calvarium to the tips of toes), with an average activity of 222 MBq (range 192 to 251 MBq).

Image data sets were ^68^Ga-PSMA-11-PET/CT with nominal administered activity 280 MBq, uptake time 60 min, and scan range from the apex to the mid-thighs. Image data sets also included NaF PET/CT with nominal administered activity 220 MBq, uptake time 60 min, and whole-body scan range. Both PET/CT scans were performed on the same PET/CT scanner within a day of each other. A total of 26 patients were analyzed in this study—14 patients in the M group and 12 patients in the N group.

Both [^68^Ga]-PSMA-11 and Na^18^F received special permission for clinical use from the Finnish Medical Evaluation Agency (FiMEA). These tracers were produced by MAP Medical Technologies Oy, Tikkakoski, Finland.

### 2.3. Image Aanalysis

The principle of the image analysis method has been previously described in [18], and the flowchart of the processing steps are presented in the Supplementary Data. Image segmentation for the evaluation of the functional volumes was performed using Maestro v6.5 (MIM Software Inc., Cleveland, OH, USA). The processing steps for image analysis were the same as those used for sodium fluoride and fluorocholine [18]. 

**Figure 1:**
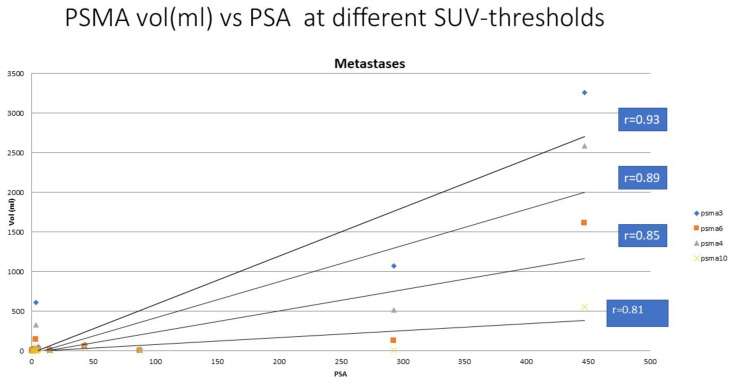
The serum PSA concentration vs. pathologic PSMA volume on ^68^Ga-PSMA PET using thresholds of 3.0, 4.0, 6.0, and 10.0 for SUV_PSMA_. The threshold value 3.0 gave the best correlation (R = 0.93), whereas the correlation coefficients were slightly weaker for threshold values of 4.0, 6.0, and 10.0, i.e., r = 0.89, 0.85, and 0.81, respectively.

The thresholds for skeleton (HU > 150) and for sclerosis (dense bone, HU > 600) were determined from the CT component of the PET/CT study and were the same as in [18]; they were based on our own experience with more than 500 PET/CT studies. The pathological NaF value (SUV > 10) was the same as that used in our analysis [18] and was similarly based on our own experience, and was similar to that of the MDACC TFL_10_ criteria, presented in the literature [19]. The pathological PSMA value (SUV > 3.0) was based on our experience in more than 300 PSMA/PET studies to indicate a pathological uptake. Thus, this value differed from that of the previous study for fluorocholine. In order to validate this, we analyzed this additional material with thresholds of 4.0, 6.0, and 10.0 for SUV_PSMA_. The summary is shown in Figure 1. 

Briefly, in this image analysis, we created eight volumes of interest (VOIs) for each patient based on the spatially coregistered PSMA-PET/CT and NaF-PET/CT scans: “Skeleton”, “Skeleton 600”, “PSMA PET 3”, “NaF PET 10”, “NaF 10 PSMA 3”, “Scl PSMA 3”, “Scl NaF 10”, and “Scl NaF 10 PSMA3” and computed their volumes (in mL).

In addition to the PET-based VOIs, we computed metrics analogous to total lesion glycolysis (TLG) in FDG PET scans, as described in [18]. For the PSMA PET-based VOIs, the product of the VOI volume and the mean SUV_bw_ in “PSMA PET 3” and “Scl PSMA 3” corresponds to the total enzyme synthesis activity (TEA) in the bone and sclerotic bone, respectively. The NaF PET-based VOIs, the product of the VOI volume and the mean SUV_bw_ in “NaF PET 10” and “Scl NaF 10”, were calculated in the same manner as in [18], corresponding to the total bone demineralization (TBA) in the bone and sclerotic bone, respectively. 

Additionally, we calculated the percentage volumes of “Skeleton 600”, “PSMA PET 3”, “NaF PET 10”, and “NaF 10 PSMA 3” with respect to “Skeleton” as we did in [18]. Similarly, the percentage volumes of “Scl PSMA 3”, “Scl NaF 10”, and “Scl NaF 10 PSMA 3” with respect to “Skeleton 600” were calculated. The mean, minimum, and maximum of the percentage volumes were presented in the program. The ratio of the mean percentage volumes between the M and N groups was calculated. A Mann–Whitney U-test was performed between the percentage volumes for the M and N groups to analyze the possible statistical differences.

## 3. Results

**Figure 2:**
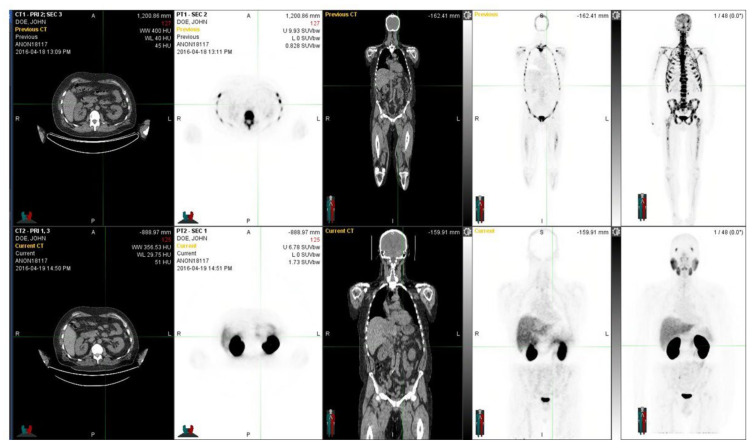
The figure demonstrates a 60-year-old prostate cancer patient diagnosed five years earlier with prostate cancer and T3bN0M0 with an initial PSA 13 and a GS 7. Despite the radical prostatectomy and hormonal therapy, he developed skeletal metastases. He was treated with chemotherapy and multiple targeted bone therapies, abiraterone, and Ra-223 radionuclide therapy. In the upper row, NaF-PET demonstrates a widespread skeletal disease in the whole skeleton, whereas in the lower row, with Ga-PSMA, fewer skeletal metastases are seen. Regions differed significantly from each other, and the PET tracers showed different overall distributions. Fluoride typically targets the cortical bone and bone formation, whereas PSMA targets active cancer cells, preferably in the bone marrow. At the time of imagings, the S-PSA was 0.35 (patient 7).

A clinical example is given in Figure 2. In this patient, NaF-PET demonstrates a more advanced skeletal disease when compared to PSMA-PET.

The patient characteristics are presented in Table 1. For comparison, we had 12 patients in the N group, i.e., prostate cancer patients without skeletal disease (Table 1).

**Figure 3:**
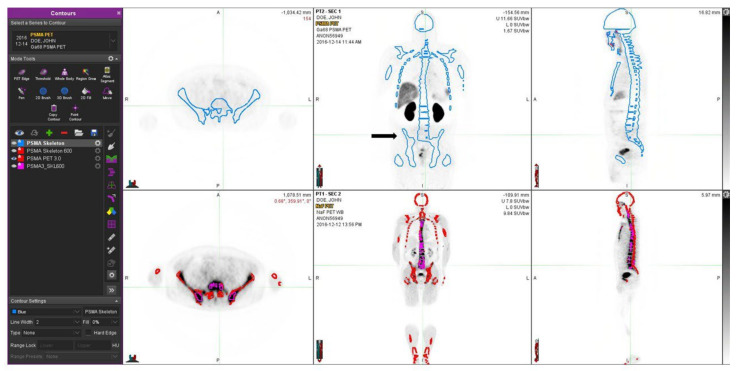
A 66-year-old patient with newly diagnosed prostate cancer and T3bN0M0 with a GS 7 is shown. He was treated with volumetric targeted radiotherapy (VMAT) and androgen deprivation. In the upper row, Ga-PSMA shows some local activity in the prostate, which is under current targeted treatment; no skeletal disease is seen. The blue contours demonstrate the thresholds based on HU > 150 on the CT component of the CT component in the PET/CT study. In the lower row, from the NaF PET study, the contours of the skeleton (HU > 150) are shown in red, and regions of sclerosis (HU > 600) are shown with purple contours. These corresponded with dense sclerotic regions on CT and were not associated with skeletal metastases. At the time of the imaging, the S-PSA was 17.1 (patient II).

One patient with no skeletal disease is shown in Figure 3. This patient has severe sclerosis in the skeleton, which may cause problems with differential diagnosis. The skeletal VOI volumes varied from 5.91 L to 11.4 L (8.77 ± 1.80 L) in patients with bone metastases (n = 14), whereas the sclerotic bone volumes varied from 1.10 L to 3.21 L (1.32 ± 0.50 L), respectively. The sclerotic bone volume varied from 0.44% to 132% of skeletal volume, as defined by PSMA-PET.

**Figure 4:**
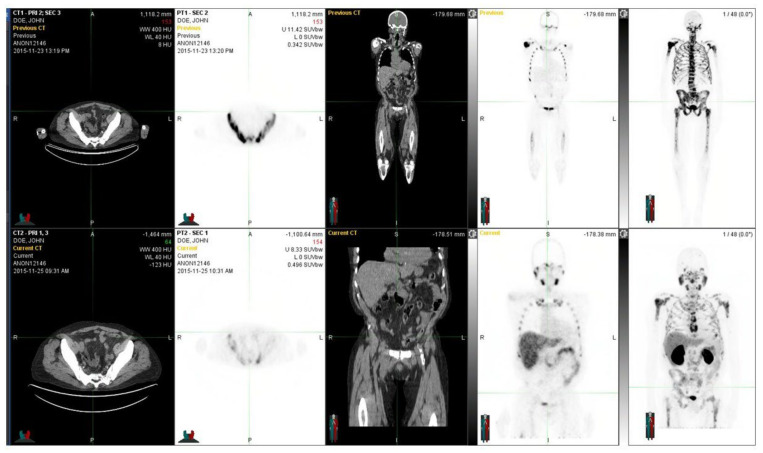
The figure demonstrates a 76-year-old patient with a T2N0M0 (GS 8) prostate cancer diagnosed more than 15 years earlier. He was originally treated with radiotherapy and androgen deprivation. After multiple relapses, an operation, additional radiotherapy, chemotherapy, and new drugs were introduced. At the time of the investigation, the widespread skeletal disease was observed both with NaF PET (upper row) and ^68^Ga-PSMA PET (lower row). There are no essential differences in their distributions. At the time of these images, the S-PSA was 293 (patient 14).

**Figure 5:**
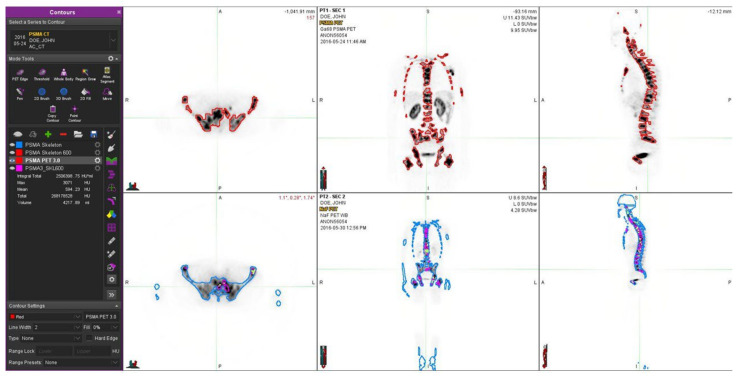
A 66-year-old patient with a T4N1M1 (GS 8) prostate cancer diagnosed three years earlier with an initial PSA of 15.0 is shown. He was originally treated with androgen deprivation and chemotherapy. After relapses, multiple new targeted bone therapies were introduced: abiraterone, Ra-223, and denosumab. At the time of the investigation, widespread skeletal disease (not very active) was observed both with NaF PET (lower row) and Ga-PSMA PET (upper row). There were essential differences in their distributions, as seen especially in the transaxial images. Fluoride typically targets the cortical bone and bone formation, whereas PSMA targets the active cancer cells, preferably in the bone marrow. Here, in the NaF images, the skeleton has better recovered from the targeted treatment, as the bone marrow still shows some activity. At the time of imaging, the S-PSA was 3.6 (patient 3).

Two more patients are shown to illustrate how an interindividual comparison of S-PSA may be misleading or helpful in the evaluation. A patient with elevated PSA (Figure 4) and with low PSA (Figure 5).

In patients with no metastases, measuring from 6.36 L to 11.6 L (8.73 ± 1.43 L, skeletal VOI) and from 1.27 L to 2.72 L (1.23 ± 0.28 L, sclerosis VOI), the sclerotic bone volume varied from 0.55% to 2.3% of skeletal volume. The TEA varied from 0.001 to 19.2 (2.21 ± 5.15) in the patients with skeletal metastases (group M) and from 0.000 to 0.194 (0.078 ± 0.053) in patients with no metastases (group N). The TBA varied from 0.981 to 20.0 (4.31 ± 6.17) in the M group metastases and from 0.000 to 2.0 (0.24 ± 0.56) PCaz in the N group (*p* < 0.0002). The pathological PSMA volume represented 0.44–132% of the sclerotic bone volume in the M group and 0.55–2.3% in the N group. The pathological NaF volume in the M group represented 0.27–68% of the sclerotic bone volume, and in the N group, it was only 0.00–6.5% of the sclerotic bone volume (*p* < 0.0003).

All the statistical differences between the M and N groups are shown in Table 2.

In Table 2, the pathological PET/CT volumes in the skeleton (when HU > 150 on CT) are presented. The pathological volumes for PSMA (SUV > 3.0), NaF (SUV > 10), and when both were pathological on PET were larger in the M group when compared to the N group, *p* < 0.0001, *p* < 0.000021, *p* < 0.00002, respectively. Sclerosis volume (HU > 600 on CT), in combination with the pathological PSMA (SUV > 3.0) volumes (*p* < 0.000022) or NaF (SUV > 10) volumes (*p* < 0.00026), were significantly larger in the M group compared to the N group. All the differences were statistically very significant (Table 2).

**Figure 6:**
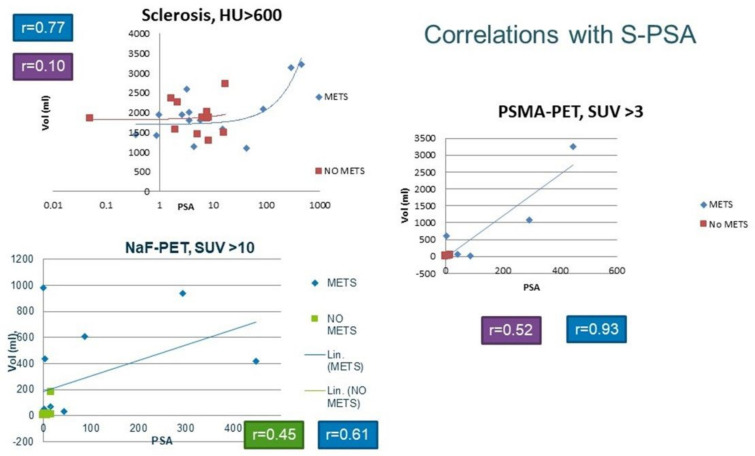
The graphs demonstrate the correlations between the total pathological PSMA volume in PET and S-PSA (on the right) and between the total pathological NaF volume in PET and S-PSA (lower left panel). In the upper left graph, the correlation between the total sclerosis volume for CT and S-PSA is shown on a logarithmic scale because of the high range of the numbers. The correlation between PSMA volume and S-PSA was strong in those patients with skeletal metastases (group M) (r = 0.93, n = 14), whereas, in patients with no bone metastases (group N), there was no correlation (r = 0.52, n = 12). The correlation was much weaker between the total pathological NaF volume in PET and S-PSA (r = 0.61, n = 14) in group M, and there was no correlation in group N (r = 0.45, n = 12). Sclerosis did not correlate with S-PSA in those patients with no bone metastases (r = 0.10, n = 12). However, there was a moderate correlation between the total sclerosis volume for CT and S-PSA (r = 0.77, n = 14) in group M.

The PET studies could easily be fixed with each other with the use of the skeletal CT because NaF-PET/CT and PSMA/PET-CT were performed on consecutive days. The skeleton did not change substantially during this time. In Figure 6, all the correlations between the imaging modalities (PSMA-PET, NaF-PET, and CT) and serum PSA concentration are shown.

In the M group, there was a strong correlation between the total pathological PSMA volumes in PET and S-PSA (r = 0.93, n = 14); in the N group, there was no correlation (r = 0.52, n = 12). The correlation in the M group was weaker between the total pathological NaF volumes in PET and S-PSA (r = 0.61, n = 14), and there was no correlation in the N group (r = 0.45, n = 12). Sclerosis did not correlate with S-PSA in those patients with no bone metastases (r = 0.10, n = 12). However, there was a moderate correlation between the total sclerosis volume in CT and S-PSA (r = 0.77, n = 14); this is shown on a logarithmic scale in Figure 6 because of the high range of the numbers.

## 4. Discussion

Earlier, we showed, in a similar analysis of skeletal metastases of prostate cancers, that CT alone cannot be used to measure the extent of active metastatic skeletal disease [18]. Then, we studied NaF and FCH and found that these radiopharmaceuticals gave complementary information about the active skeletal metastases and even improved the primary diagnosis and secondary staging [18]. Here, we analyzed PSMA-PET, NaF-PET, and CT contemporaneously in advanced metastatic skeletal disease in a larger patient population. 

This is the only study in the literature where NaF and PSMA-PET have been studied in the same patients within two days. In this analysis, we found out how, at the voxel level (4 mm × 4 mm × 4 mm), the tracer uptakes differed or overlapped. These datasets are huge, with more than several hundred thousand (10^5^) voxels per patient. The PET screening of these patients was part of their routine program; no other selection criteria were applied. Besides the small number of patients, this was a limiting factor. The large volumes of radiomic features compensated for this, indicating that there was a lot of data at the voxel level. 

PSMA-PET has become more important and attractive because new Ga-68-labelled PSMA compounds have received marketing approval (Locametz^®^ and Illuccix^®^). Therefore, it is important to understand if the earlier findings are valid with ^68^Ga]-PSMA-11-PET.

There was no statistical difference (*p* > 0.55, nonsignificant) in the amount of sclerosis between the M and N groups; the sclerotic bone volume was 1320 ± 501 mL, as defined by PSMA-PET in the patients with bone metastases (M), and 1231 ± 278 mL in the prostate cancer patients without skeletal metastases (N) (Table 2). Because of the similarity in sclerosis volumes in the M and N groups, these groups could be compared with each other. 

We analyzed the pathological PET/CT volumes inside the skeleton (as defined HU > 150 on CT) for PSMA (SUV > 3.0), NaF (SUV > 10), and when NaF and PSMA were contemporaneously pathological. These findings were statistically extremely significant between the M and N groups: pathological skeletal PSMA volume: *p* < 0.0001, pathologic skeletal NaF volume: *p* < 0.000021, and pathological contemporaneous PSMA & NaF volume: *p* < 0.00002, respectively (Table 2). The volumes were larger in those patients with metastases (M) when compared to the N group. Sclerosis from CT (as defined HU > 600) in combination with the pathological PSMA and NaF volumes was also larger in the M group than in the N group. Both these differences were statistically significant, as shown in Table 2, with *p* < 0.000022 for the sclerotic PSMA volume and *p* < 0.00026 for the sclerotic NaF volume. These findings confirm that these two PET methods, PSMA and NaF, measure different phenomena. Even though our findings are statistically very significant, the small patient number may have an effect on the results.

We also found that our new parameters (analogous to TLG: total lesion glycolysis for ^18^FDG-PET) were useful for total enzyme activity for PSMA (TEA) and total accelerated osteoblastic activity (total bone demineralization) activity for NaF (TBA) [17,18]. Unfortunately, our patient population is too small to judge if these parameters have prognostic value.

The enzymatic activity of PSMA is considered part of the catalytic zinc metallopeptidase family M28, and its enzymatic behavior is dualistic. Although PSMA is encoded by folate hydrolase 1 gene (FOLH1), which is associated with a major role in folate uptake, PSMA usually acts as a glutamate carboxypeptidase II (GCPII) in different tissues and hydrolyzes N-acetylaspartyl-glutamate (NAAG) [20,21]. Originally, derivatives based on NAAG were developed as PSMA inhibitors and evaluated for their targeting characteristics. The compound we used here, ^68^Ga-PSMA-11, is the most investigated PET agent for imaging prostate cancer. Afshar-Oromieh et al. published the first clinical evaluation of ^68^Ga-PSMA-11 in prostate cancer in 2012 [22]. ^68^Ga-PSMA-11 detects recurrent and metastatic prostate cancer by binding to the extracellular domain of PSMA. Since then, multiple studies have been conducted to compare ^68^Ga -PSMA-11 with other conventional PET tracers for imaging patients with prostate cancer. The PSMA targetors (^68^Ga-PSMA-11, ^18^F-PSMA-1007, and ^18^F-DCFPyl) turned out to be better than conventional PET tracers in the meta-analysis [8].

Despite the small number of patients (26 total), in almost every patient we found small volumes of PSMA and NaF, which did not overlap. Some findings are seen in patient examples, especially in the transaxial images in Figure 3 and Figure 5. This dual tracer method with PSMA/NaF improves routine diagnostics because it allows for the localization of bone marrow, cortical bone, and activity at their interface. Bone metastases are formed by hematogenic spreading; therefore, the differential diagnosis of active disease, either in bone marrow and/or in cortical bone, is a useful piece of information. However, we are not yet sure about the quality and alignment of the contemporaneous studies; therefore, with the help of the rigid skeleton to two PET studies, this can be fixed for volumetric analysis.

**Figure 7:**
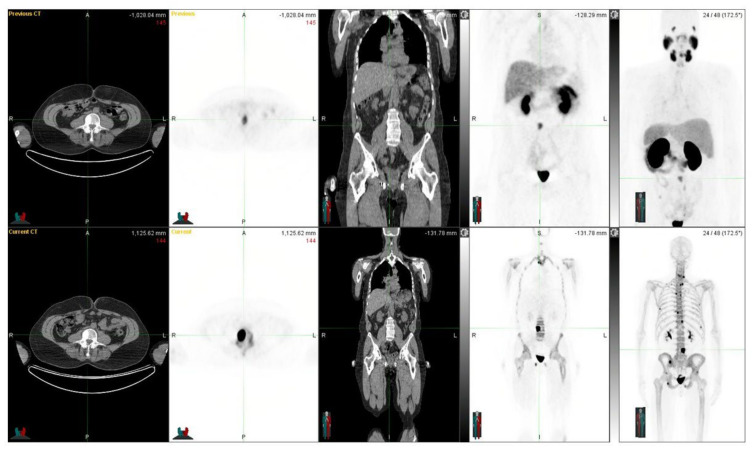
Demonstrates a situation where ^18^F-NaF PET/CT and ^68^Ga-PSMA-11 PET/CT differ significantly from each other.

Figure 7 shows a 54-year-old patient with a T4N1M1 (GS 9) prostate cancer diagnosed four years earlier, with an initial PSA of 0.92. He was originally treated with radical prostatectomy, androgen deprivation, and radiotherapy. After relapses, bone therapies were introduced: targeted radiotherapy and Ra-223. At the time of the investigation, widespread skeletal disease (yet not very active) was observed extensively with NaF PET (lower row) and only weakly with Ga-PSMA PET (upper row). Ga-PSMA PET shows only those lesions in the left iliac crest and lumbar vertebra L4 (SUVmax 7.5), whereas NaF-PET shows multiple skeletal lesions in almost all bones with SUVmax ad 107. The threshold values for the pathological findings were 3.0 for Ga-PSMA and 10.0 for NaF (patient 10).

We have shown that, in patients with bone metastases, there was a strong correlation between the total pathological PSMA volume in PET and S-PSA (r = 0.93, n = 14). However, there was a moderate correlation between the total sclerosis volume for CT and S-PSA (r = 0.77, n = 14); actually, any PSMA threshold between 4.0–10.0 gives a better correlation (r = 0.81–0.89) than CT. 

Bone metastases in prostate cancer are considered osteoblastic. However, other types of metastases occur. In a recent study of 963 prostate cancer patients, 188 patients (19.5%) demonstrated skeletal metastases [23]. Out of these, 70.7% demonstrated osteoblastic type metastases, whereas intramedullary metastases were found in 51.6% of the patients, and osteolytic metastases were detected in 36 patients (19.2%).

In our institution, two prostate cancer PET studies have been used routinely for diagnosis. This practice had to be synchronized with radiopharmaceutical production because most of the patients traveled long distances, even from abroad, and the hospital time was limited. This meant that our prostate cancer patients had different first-line, second-line, third-line, etc., treatments. When assessing biological response, e.g., with S-PSA, it is important to understand if the prostate is present or not and how it has been removed or not removed at all. Here, we measured the skeletal disease with two radiopharmaceuticals, and it is obvious that the treatment may have an effect on the development of bone metastases. Metastases formation occurs due to hematogenic spreading; here, it is related to the conditions in the bone marrow, such as the use of bone marrow stimulating agents. New bone targeting agents may play a pivotal role in metastasis formation on site in the cortical bone.

Fortunately, we did not have any exceptions for PET scanning in this study. Today, dual-tracer studies may also include PSMA-PET and FDG-PET because FDG has been used to exclude those patients who do not respond to PSMA-targeted therapies [24]. With the NaF and PSMA approach, we could select patients who benefit from bone-targeting therapies, such as Ra-223 (Xofigo^®^) or denosumab. Also, the first PSMA-targeted therapeutic compound (Lu-177-vipivotide tetraxetan, Pluvicto^®^, PSMA-617) received approval from the Food and Drug Administration this year. Additionally, the kit formulations for ^68^Ga-labelling, Locametz^®^, and Illuccix^®^ have recently received marketing authorization. In an Australian trial, FDG-PET was used to exclude those patients with poor prognosis trials [25]. Thus, the dual-tracer PET method is an acceptable diagnostic tool for those patients who plan on using targeted radionuclide therapies for treatment. The PET tracer costs are minimal when compared to the costs of therapy, Lu-177-PSMA (Pluvicto^®^) costs approximately USD 50,000 per cycle; PET studies cost less than 10% of that. In the VISION [26] and TheraP trials [25], the patients were excluded according to strict criteria; actually, the response followed that the more the patients were excluded, the better the outcome: TheraP excluded 28% of the patients, and the response rate was 66%, whereas VISION excluded 12% of the patients, and the response rate was 46%, respectively.

Sodium fluoride has turned out to be a useful follow-up for PCa with skeletal metastases [27,28], and, in our hands, it even suits the routine quantification of treatment responses [28,29]. We have developed quantitative criteria for bone-targeted therapies [30]. This is important because the high uptake of ^18^F-NaF reflects bone reactions to skeletal disease, not necessarily the reactions to cancer. Therefore, positive findings with ^18^F-NaF PET/CT may be due to both benign and malignant bone disorders. Despite this, sodium fluoride is included in nationwide recommendations in the assessment of the skeletal metastases of prostate cancer [12]. Only a little interobserver variability was noticed in an analysis of 219 patients, out of which 129 patients were scanned for primary staging, 67 for biochemical recurrence, and 23 for metastatic castration-resistant prostate cancer [31]. PSMA compounds (^18^F-DCFPyL, ^18^F-sodium fluoride (^18^F-NaF), and ^18^F-FDG PET/CT) have been compared prospectively in men with metastatic prostate cancer [32]. The PSMA compound ^18^F-DCFPyL is most versatile for detecting metastatic PCa, whereas ^8^F-NaF detects more bone metastases [32]. Similar observations were made for the PSMA compound ^18^F-DCFBC [33]. However, PSMA compounds are better at localizing skeletal disease than bone scintigraphy and are sometimes better than bone-targeting therapeutic agents [34,35]. We know from the global WARMTH Study with Lu-177-PSMA-617 (Pluvicto^®^) that the presence of skeletal metastases will worsen the prognosis significantly [36]. On the other hand, we know that NaF can be used to select patients for Ra-223 treatment regarding skeletal disease [37]. Similarly, here, we were able to select patients who had poor responses to Lu-177-PSMA. For example, with predefined criteria for skeletal disease volume, we could improve the outcome of Lu-177-PSMA-617-treatment or apply tandem treatments (Xofigo^®^ + Pluvicto^®^) to a selected patient group.

We had almost identical results in a treatment-naive (Figure 3) and in a heavily treated patient (Figure 4); both patients had an aggressive T3b/T4 disease, and PSMA and NaF gave complementary information, even though the latter patient had targeted bone therapies. There are multiple PET radiopharmaceuticals to choose from in prostate cancer [38]. Most of them have got marketing authorization, besides the earlier mentioned gozetotide (Locametz^®^ and Illuccix^®^) e.g., fluciclovine (Axumin^®^) and piflufolastat (Pylarify^®^) are commercially available. The combination of NaF-PSMA we used here for volumetric radiomics analysis in the skeleton turned out to be exceptional. However, other alternatives should be tested in clinical trials because the behavior of various PSMA compounds in the skeleton is not yet fully understood. PSMA is a central new target [33,34] because PSMA-targeted treatments offer excellent theragnostic potential and are competitive with all of the current therapies for prostate cancer [39]. However, patients who have the presence of skeletal disease have a less favorable prognosis. These patients could respond to alternative concomitant bone-targeting therapies. In order to identify these patients, this dual-tracer method could be an excellent approach.

## 5. Conclusions

We can confirm that our previous finding from the FCH-NaF study is valid here as well: CT alone is not well-suited for measuring the extent of active skeletal disease in advanced prostate cancer. NaF and PSMA measure different phenomena, and both suit the evaluation of active skeletal disease and actually provide complementary information. This opens new possibilities for detailed treatment planning for polyclonal bone-dominant disease. Furthermore, PSMA correlates positively with serum PSA concentration. A Cut-off value 3.0 for SUV_PSMA_ gave the best correlation in the presenting active metastatic skeletal disease.

## Figures and Tables

**Table 1:** Patient characteristics are shown in the table: age, Gleason score (GS), TNM classification, time of diagnosis, initial S-PSA, previous treatments, skeletal therapies, and S-PSA at the time of analysis. Abbreviations: A—abiraterone, ADT—androgen deprivation, Ch—chemotherapy, D—denosumab, IT-immunotherapy, Ra—radium-223, RP = radical prostatectomy, Rx—radiotherapy, Sm—Sm-153, and Z—zolendronate. Arabic numbers refer to the M group, and Roman numbers to the N group.

No/Age	Gleason Score	TNM	Time of dgn	iPSA	Previous Treatments	Bone Therapies	PSA at PET [µg/l]
1/54	9 (5 + 4)	T4N1M1	VII-2013	10	RP, ADT, Rx	Ra	0.92
2/66	7 (4 + 3)	T3aN1M0	VIII-08	7.7	Rx, ADT, Ch, IT	Sm, D, A	87.5
3/78	6 (3 + 3)	T2cN0M0	VII-11	15	Rx, ADT, Ch	D, A, Ra	3.6
4/54	7 (4 + 3)	T3N1M0	VI-09	21	Rx, ADT, Ch	Z, D	4.9
5/61	7 (4 + 3)	T2aN0MX	VII-11	n.a.	RP, ADT, Ch	Z, Ra, A	1.39
6/60	7 (3 + 4)	T3bN0M0	VI-10	13	RP, ADT, Ch	Ra, A	0.35
7/68	7 (4 + 3)	T3bN0M0	III-15	38	RP, ADT, Ch	A, Ra	15
8/79	7 (3 + 4)	T3N0M0	III-06	6.7	RP, ADT, Rx, Ch	Z, Sm	0.98
9/77	9 (4 + 5)	T4NXM1	II-04	490	ADT, Rx, Ch	Z	4,39
10/66	8 (4 + 4)	T4N1M1	VI-13	15	ADT, Ch	A, Ra, D	447
11/57	7 (3 + 4)	T2N1M1	I-13	968	ADT, Rx, Ch	D, Sm	3.6
12/74	5	T1cN0M0	II-96	16	RP, ADT, Rx, Ch	D, A	43.1
13/57	8 (4 + 4)	T3aN0M0	III-14	58	Rx, ADT		2.6
14/76	8 (4 + 4)	T2N0M0	VII-99	n.a.	Rx, ADT, TURP, Ch	Z	293
I/77	8 (4 + 4)	T3N0M0	VIII-16	0.3			8.29
II/65	7 (3 + 4)	T3bN0M0	XI-16	17.1	Rx		17.1
III/65	6 (3 + 3)	T1cN0M0	II-16	6.3	Rx		6.3
IV/83	9 (4 + 5)	T3N1M1	XI-11	26.7	ADT, Rx, Ch, IT	A	16.3
V/63	7 (4 + 3)	T4N0M0	I-08	28	RP, Rx, ADT	Z	1.98
VI/68	7 (4 + 3)	T3N0M0	V-09	8	RP, Rx, ADT	Z, Sm	1.7
VII/74	9 (5 + 4)	T3N0M0	XII-13	0.48	ADT, Rx		<0.05
VIII/69	7 (4 + 3)	T3N0M0	II I-10	22	Rx, ADT	Z	2.05
IX/77	7 (4 + 3)	T1N0M0	X-16	8.3	TURP, Rx		2.23
X/50	7 (3 + 4)	T1N0M0	XI-16	8.5	Rx		8.5
XI/81	6 (3 + 3)	T2bN0M0	XII-97	10.5	RP, Rx, ADT		7.94
XII/50	7 (3 + 4)	T4N0M0	I-08	36	RP, Rx		7.81

**Table 2:** Results. From the left, the pathological PET/CT volumes are shown in the skeleton (HU > 150 on CT) for PSMA (SUV > 3), NaF (SUV > 10), and when both are pathological. The sclerosis volume from CT (HU > 600) is shown next (in the middle). Simultaneous sclerosis and pathological volumes for both PSMA and/or NaF are shown on the right. The volumes are shown in the M group (METS) and the N group (NO METS). The amount of sclerosis does not statistically differ between these patient groups (*p* < 0.55). All other pathological volumes differ significantly (statistically) from each other between the M and N groups. Statistical hypothesis: Mann–Whitney U-test for noninferiority.

	PSMA > 3	NaF > 10	PSMA >3 & NaF > 10	HU > 600	PSMA > 3& HU > 600	NaF > 10 & HU > 600	PSMA > 3 & NaF > 10 & HU > 600
METS (n = 14)	373 ± 885	262 ± 354	88.9 ± 170	1320 ± 501	327 ± 1120	54.0 ±110	19.9 ± 48.5
NO METS (n = 12)	19.2 ± 8.68	21.0 ± 49.3	0.050 ± 0.15	1231 ± 278	7.02 ± 3.53	2.29 ± 2.13	0.00 ± 0.00
*p*<	0.0010	0.000021	0.000020	0.55	0.000022	0.00026	0.000003

## Data Availability

Not applicable.

## Supplementary Materials

The following supporting information can be downloaded at: https://www.mdpi.com/article/10.3390/life12121977/s1, Supplementary Data.

## References

[1] Lange P.H., Vessella R.L. (1999). Mechanisms, hypotheses and questions regarding prostate cancer micrometastases to bone. Cancer Metastasis Rev..

[2] Alva A., Hussain M. (2013). The Changing Natural History of Metastatic Prostate Cancer. Cancer J..

[3] Scher H.I., Sawyers C.L. (2005). Biology of progressive, castration-resistant prostate cancer: Directed therapies targeting the androgen receptor signaling axis. J. Clin. Oncol..

[4] Saad F., Hotte S.J. (2010). Guidelines for the management of castrate-resistant prostate cancer. Can. Urol. Assoc. J..

[5] Sathiakumar N., Delzell E., Morrisey M.A., Falkson C., Yong M., Chia V., Blackburn J., Arora T., Kilgore M.L. (2011). Mortality following bone metastasis and skeletal-related events among men with prostate cancer: A population-based analysis of US Medicare beneficiaries, 1999–2006. Prostate Cancer Prostatic Dis..

[6] Haberkorn U., Eder M., Kopka K., Babich J.W., Eisenhut M. (2016). New Strategies in Prostate Cancer: Prostate-Specific Membrane Antigen (PSMA) Ligands for Diagnosis and Therapy. Clin. Cancer Res..

[7] Luining W.I., Cysouw M.C.F., Meijer D., Hendrikse N.H., Boellaard R., Vis A.N., Oprea-Lager D.E. (2022). Targeting PSMA Revolutionizes the Role of Nuclear Medicine in Diagnosis and Treatment of Prostate Cancer. Cancers.

[8] Alberts I.L., Seide S.E., Mingels C., Bohn K.P., Shi K., Zacho H.D., Rominger A., Afshar-Oromieh A. (2021). Comparing the diagnostic performance of radiotracers in recurrent prostate cancer: A systematic review and network meta-analysis. Eur. J. Nucl. Med. Mol. Imaging.

[9] Von Eyben F.E., Kairemo K. (2014). Meta-analysis of 11C-choline and 18F-choline PET/CT for management of patients with prostate cancer. Nucl. Med. Commun..

[10] Alongi P., Laudicella R., Lanzafame H., Farolfi A., Mapelli P., Picchio M., Burger I.A., Iagaru A., Minutoli F., Evangelista L. (2022). PSMA and Choline PET for the Assessment of Response to Therapy and Survival Outcomes in Prostate Cancer Patients: A Systematic Review from the Literature. Cancers.

[11] Segall G., Delbeke D., Stabin M.G., Even-Sapir E., Fair J., Sajdak R., Smith G.T. (2010). SNM practice guideline for sodium 18F-fluoride PET/CT bone scans 1.0. J. Nucl. Med..

[12] Mogensen A.W., Petersen L.J., Torp-Pedersen C., Nørgaard M., Pank M.T., Zacho H.D. (2022). Use of ^18^F-NaF PET in the staging of skeletal metastases of newly diagnosed, high-risk prostate cancer patients: A nationwide cohort study. BMJ Open.

[13] Etchebehere E.C., Araujo J.C., Fox P.S., Swanston N.M., Macapinlac H.A., Rohren E.M. (2015). Prognostic Factors in Patients Treated with223Ra: The Role of Skeletal Tumor Burden on Baseline 18F-Fluoride PET/CT in Predicting Overall Survival. J. Nucl. Med..

[14] Apolo A.B., Lindenberg L., Shih J.H., Mena E., Kim J.W., Park J.C., Alikhani A., McKinney Y.Y., Weaver J., Turkbey B. (2016). Prospective Study Evaluating Na^18^F PET/CT in Predicting Clinical Outcomes and Survival in Advanced Prostate Cancer. J. Nucl. Med..

[15] Joensuu T., Joensuu G., Kairemo K., Kiljunen T., Riener M., Aaltonen A., Ala-Opas M., Kangasmäki A., Alanko T., Taipale L. (2016). Multimodal Primary Treatment of Metastatic Prostate Cancer with Androgen Deprivation and Radiation. Anticancer Res..

[16] Ahmadzadehfar H., Rahbar K., Baum R.P., Seifert R., Kessel K., Bögemann M., Kulkarni H.R., Zhang J., Gerke C., Fimmers R. (2021). Prior therapies as prognostic factors of overall survival in metastatic castration-resistant prostate cancer patients treated with [^177^Lu]Lu-PSMA-617. A WARMTH multicenter study (the 617 trial). Eur. J. Nucl. Med. Mol. Imaging.

[17] Kairemo K., Kangasmäki A., Kappadath S.C., Joensuu T., Macapinlac H.A. (2017). A retrospective analysis of sodium fluoride Na18F-PET/CT and 68Ga-PSMA-11 PET/CT in the skeletal metastases in metastatic prostate cancer using a volumetric 3-D analysis. J. Nucl. Med..

[18] Kairemo K., Kappadath S.C., Joensuu T., Macapinlac H.A. (2021). A Retrospective Comparative Study of Sodium Fluoride (NaF-18)-PET/CT and Fluorocholine (F-18-CH) PET/CT in the Evaluation of Skeletal Metastases in Metastatic Prostate Cancer Using a Volumetric 3-D Radiomics Analysis. Diagnostics.

[19] Rohren E.M., Etchebehere E.C., Araujo J.C., Hobbs B.P., Swanson N.M., Everding M., Moody T., Macapinlac A. (2015). Deter-mination of Skeletal Tumor Burden on 18F-Fluoride PET/CT. J. Nucl. Med..

[20] Rawlings N.D., Barrett A.J. (1997). Structure of membrane glutamate carboxypeptidase. Biochim. Biophys. Acta.

[21] O’Keefe D.S., Bacich D.J., Huang S.S., Heston W.D.W. (2018). A Perspective on the evolving story of PSMA biology, PSMA-based imaging, and endoradiotherapeutic strategies. J. Nucl. Med..

[22] Afshar-Oromieh A., Haberkorn U., Eder M., Eisenhut M., Zechmann C. (2012). [68Ga]-Gallium-labelled PSMA ligand as superior PET tracer for the diagnosis of prostate cancer: Comparison with 18F-FECH. Eur. J. Nucl. Med. Mol. Imaging.

[23] Kesler M., Kerzhner K., Druckmann I., Kuten J., Levine C., Sarid D., Keizman D., Yossepowitch O., Even-Sapir E. (2021). Staging ^68^Ga-PSMA PET/CT in 963 consecutive patients with newly diagnosed prostate cancer: Incidence and characterization of skeletal involvement. Eur. J. Pediatr..

[24] Hofman M.S., Emmett L., Violet J., Zhang A., Lawrence N.J., Stockler M., Davis I.D. (2019). TheraP: A randomized phase 2 trial of (177) Lu-PSMA-617 theranostic treatment vs cabazitaxel in progressive metastatic castration-resistant prostate cancer (Clinical Trial Protocol ANZUP 1603). BJU Int..

[25] Hofman M.S., Emmett L., Sandhu S., Iravani A., Joshua A.M., Goh J.C., Pattison D.A., Tan T.H., Kirkwood I.D., Ng S. (2021). [177Lu]Lu-PSMA-617 versus cabazitaxel in patients with metastatic castration-resistant prostate cancer (TheraP): A random-ised, open-label, phase 2 trial. Lancet.

[26] Sartor O., de Bono J., Chi K.N., Fizazi K., Herrmann K., Rahbar K., Tagawa S.T., Nordquist L.T., Vaishampayan N., El-Haddad G. (2021). Lutetium-177-PSMA-617 for Metastatic Castration-Resistant Prostate Cancer. N. Engl. J. Med..

[27] Even-Sapir E., Metser U., Mishani E., Lievshitz G., Lerman H., Leibovitch I. (2006). The detection of bone metastases in patients with high-risk prostate cancer: 99mTc-MDP Planar bone scintigraphy, single- and multi-field-of-view SPECT, 18F-fluoride PET, and 18F-fluoride PET/CT. J. Nucl. Med..

[28] Kairemo K., Macapinlac H.A., Kairemo K., Macapinlac H. (2020). Sodium Fluoride Imaging in Oncology. Sodium Fluoride PET/CT in Clinical Use.

[29] Kairemo K.J.A., Joensuu T. (2015). Radium-223-Dichloride in Castration Resistant Metastatic Prostate Cancer—Preliminary Results of the Response Evaluation Using F-18-Fluoride PET/CT. Diagnostics.

[30] Kairemo K., Milton D.R., Etchebehere E., Rohren E.M., Macapinlac H.A. (2018). Final Outcome of 223Ra-therapy and the Role of 18F-fluoride-PET in Response Evaluation in Metastatic Castration-resistant Prostate Cancer—A Single Institution Experience. Curr. Radiopharm..

[31] Zacho H.D., Fonager R.F., Nielsen J.B., Haarmark C., Hendel H.W., Johansen M.B., Mortensen J.C., Petersen L.J. (2020). Observer Agreement and Accuracy of ^18^F-Sodium Fluoride PET/CT in the Diagnosis of Bone Metastases in Prostate Cancer. J. Nucl. Med..

[32] Fourquet A., Rosenberg A., Mena E., Shih J.J., Turkbey B., Blain M., Bergvall E., Lin F., Adler S., Lim I. (2022). Comparison of 18 F-DCFPyL, 18 F-NaF, and 18 F-FDG PET/CT I,n a Prospective Cohort of Men with Metastatic Prostate Cancer. J. Nucl. Med..

[33] Harmon S.A., Bergvall E., Mena E., Shih J.H., Adler S., McKinney Y., Mehralivand S., Citrin D.E., Couvillon A., Madan R.A. (2018). A Prospective Comparison of ^18^F-Sodium Fluoride PET/CT and PSMA-Targeted ^18^F-DCFBC PET/CT in Metastatic Prostate Cancer. J. Nucl. Med..

[34] Lawal I.O., Mokoala K.M.G., Mahapane J., Kleyhans J., Meckel M., Vorster M., Ebenhan T., Rösch F., Sathekge M.M. (2020). A prospective intra-individual comparison of [^68^Ga]Ga-PSMA-11 PET/CT, [^68^Ga]Ga-NODAGAZOL PET/CT, and [^99^mTc]Tc-MDP bone scintigraphy for radionuclide imaging of prostate cancer skeletal metastases. Eur. J. Nucl. Med. Mol. Imaging.

[35] Pepe P., Pennisi M. (2022). Should ^68^Ga-PSMA PET/CT Replace CT and Bone Scan in Clinical Staging of High-risk Prostate Cancer?. Anticancer Res..

[36] Ahmadzadehfar H., Matern R., Baum R.P., Seifert R., Kessel K., Bögemann M., Kratochwil C., Rathke H., Ilhan H., Svirydenka H. (2021). The impact of the extent of the bone involvement on overall survival and toxicity in mCRPC patients receiving [^177^Lu]Lu-PSMA-617: A WARMTH multicentre study. Eur J. Nucl. Med. Mol. Imaging.

[37] Oyen W., Sundram F., Haug A.R., Kairemo K., Lewington V., Mäenpää H., Mortensen J., Mottaghy F., Virgolini I., O’Sullivan J.M. (2015). Radium-223 Dichloride (Ra-223) for the Treatment of Metastatic Castration-resistant Prostate Cancer: Optimizing Clinical Practice in Nuclear Medicine Centers. J. Onco Pathol..

[38] Kairemo K.J.A. (2017). PET/Computed Tomography for Radiation Therapy Planning of Prostate Cancer. PET Clin..

[39] Von Eyben F.E., Kairemo K., Paller C., Hoffmann M.A., Paganelli G., Virgolini I., Roviello G. (2021). ^177^Lu-PSMA Radioligand Therapy Is Favorable as Third-Line Treatment of Patients with Metastatic Castration-Resistant Prostate Cancer. A Systematic Review and Network Meta-Analysis of Randomized Controlled Trials. Biomedicines.

