# Supplementary material for: A Retrospective Comparative Study of Sodium Fluoride Na18F-PET/CT and 68Ga-PSMA-11 PET/CT in the Bone Metastases of Prostate Cancer Using a Volumetric 3-D Radiomic Analysis
Source: Life (Basel). 2022 Nov 25;12(12):1977. doi: 10.3390/life12121977 (PMC9788581; doi:10.3390/life12121977)
Supplement: Supplementary file 1 [file life-12-01977-s001.zip › life-2019676-supplementary.pdf]

Supplementary Data

The image analysis processing steps were as follows:

1. The skeleton VOI was segmented by selecting voxels with HU > 150 on the CT scans from Ga-68-PSMA-11- (PSMA-PET) and NaF PET/CT (NaF-PET) scans.

2. A rigid registration of the two skeleton VOIs from [Ga-68]-PSMA-11-PET/CT and NaF PET/CT scans was performed. Now the two PET and CT scans are spatially co-registered in a single frame of reference. The skeleton VOI was labeled as "Skeleton" for figures.

3. A subset VOI of sclerotic (pathologic) bone was created by selecting voxels with HU > 600 within the skeleton VOI—labeled as "Skeleton 600".

4. A PET based VOI labeled " PSMA PET 3" was created from the overlap of voxels with [Ga-68]-PSMA-11-- PET SUV_{bw} > 3.0 and the "Skeleton" VOI.

5. A PET based VOI labeled "NaF PET 10" was created from the overlap of voxels with NaF PET SUV_{bw} > 10 and the "Skeleton" VOI.

6. A PET based VOI labeled "NaF 10 PSMA 3" was the overlap of "PSMA PET 3" VOI and "NaF PET 10" VOI.

7. A PET based VOI labeled "Scl PSMA 3" was the overlap of " PSMA PET 3" VOI and "Skeleton 600" VOI.

8. A PET based VOI labeled "Scl NaF 10" was the overlap of "NaF PET 10" VOI and "Skeleton 600" VOI.

9. A PET based VOI labeled "Scl NaF 10 PSMA 3" was the overlap of "Scl PSMA 3" VOI and "Scl NaF 10" VOI.

As result of this, eight Volumes of interest (VOIs) were created for each patient based on spatially co-registered PSMAPET/CT and NaF PET/CT scans; namely, "Skeleton", "Skeleton 600", " PSMA PET 3", "NaF PET 10", "NaF 10 PSMA 3", "Scl PSMA 3", "Scl NaF 10", and "Scl NaF 10 PSMA3" and their volumes could be computed (in ml).
